# The economic impact of living with a rare disease for children and their families: a scoping review protocol

**DOI:** 10.12688/hrbopenres.13765.1

**Published:** 2023-08-31

**Authors:** Niamh Buckle, Orla Doyle, Naonori Kodate, Suja Somanadhan

**Affiliations:** 1 School of Nursing, Midwifery and Health Systems, University College Dublin, Dublin, Leinster, D04 V1W8, Ireland; 2 School of Economics, University College Dublin, Dublin, Leinster, D04 N9Y1, Ireland; 3 School of Social Policy, Social Work and Social Justice, University College Dublin, Dublin, Leinster, D04 N9Y1, Ireland; 4 UCD Centre for Interdisciplinary Research, Education and Innovation in Health Systems (UCD IRIS), University College Dublin, Dublin, Leinster, Ireland

**Keywords:** Child, cost-of-illness, economic evaluation, family, financial hardship, orphan diseases, rare diseases, ultra-rare diseases

## Abstract

**Background:** Rare diseases are an often chronic, progressive and life-limiting group of conditions affecting more than 30 million people in Europe. These diseases are associated with significant direct and indirect costs to a spectrum of stakeholders, ranging from individuals and their families to society overall. Further quantitative research on the economic cost for children and their families living with a rare disease is required as there is little known on this topic. This scoping review aims to document the extent and type of evidence on the economic impacts of living with a rare disease for children and their families.

**Methods:** This scoping review will follow the PRISMA-ScR and Joanna Briggs Institute guidelines and follow the six-stage methodology for scoping reviews: (1) identifying the research question, (2) identifying relevant studies, (3) study selection, (4) charting the data, (5) collating, summarising and reporting results and (6) knowledge user consultation. Key inclusion criteria have been developed according to the Population-Concept-Context (PCC) framework. The databases EconLit, ABI/Inform, MEDLINE, PubMed, CINAHL, and Scopus will be searched for possible articles for inclusion. Two independent reviewers will screen titles and abstracts of potential articles using a dual review process to ensure all relevant studies are included. All included articles will be assessed using a validated quality appraisal tool. A panel of patient and public involvement representatives experiencing rare diseases and knowledge users will validate the review results.

**Conclusions:** This scoping review will map the current literature on the economic impact of paediatric rare diseases to understand how these impacts affect children living with rare diseases and their families. This evidence has the potential to influence policy and future research in this area and will support the future development of a cost-minimal intervention prototype to address the economic impact for families as part of this doctoral project.

## Introduction

The collective incidence of rare diseases contributes to a significant global population that belies its ‘rare’ title. It is estimated that there are between 6,000 and 8,000 known rare diseases ( European Commission, n.d.; Kruse *et al*., 2022) that affect 3.5%-5.9% of the global population ( Nguengang Wakap *et al*., 2020). In Europe, a rare disease is one that affects less than one in 2,000 people, resulting in a rare disease population of approximately 30 million people on the continent ( Kole *et al*., 2021). In the United States, the definition of a rare disease is one that affects less than 200,000 people; it is estimated that 10% of the US population are living with a rare disease ( National Organisations for Rare Disorders (NORD), 2019). Rare diseases are often incurable, complex and are associated with a high social and economic burden that reaches from the patient to society overall ( García-Pérez *et al*., 2021; Somanadhan *et al*., 2023a)

Approximately 70% of rare diseases start in childhood, and early diagnosis and treatment is critical to prevent disease progression and avoid adverse health outcomes, including disability and death ( Morton *et al*., 2022). Eighty percent of rare diseases have a genetic component and therefore treatment is often limited or costly, and 95% of rare diseases lack an approved treatment ( Somanadhan *et al*., 2023b). This brings challenges regarding funding for treatment research and development and treatment accessibility, as orphan drugs, which are developed specifically for rare diseases, can cost up to five times more than non-orphan drugs ( Chambers *et al*., 2020). Few rare diseases have orphan drugs, contributing to a large unmet need ( Rodriguez-Monguio *et al.*, 2017). Other treatments for rare diseases, such as gene therapy, which involves modification of genes to treat, prevent or cure an illness or disease ( White, 2019), similarly come at a high cost and are limited. It is important that treatment developments in this field balance treatment accessibility for patients, encouragement of innovation and development for researchers, clinicians and pharmaceutical companies, and creation of benefits for population health for policymakers to ensure the continued advancement and availability of treatments for the rare disease population ( European Medicines Agency, 2023; UN Committee on Economic, Social and Cultural Rights (CESCR), 2000).

The lack of funding for orphan drug development stems from the limited market potential of rare diseases, as developing drugs for limited patient groups is unlikely to be cost effective and be sustainable for pharmaceutical companies ( Iizuka & Uchida, 2017). However, this has both human and economic costs. High prices and reduced access to treatment can cause increased health burden and premature death for patients with rare diseases; lack of treatment access also reduces rare disease patients’ capacity to engage in society independently, incurring costs such as loss of income tax, absenteeism and presenteeism of carers, and potential increased healthcare costs due to the consequences of poor health ( Hyry *et al*., 2013). The United Nations Resolution on Persons Living with a Rare Disease and their Families, ratified in 2021, calls on member states to cover quality essential health products, health services and quality, safe, effective, affordable and essential medicines, diagnostics, and health technologies for people living with a rare disease ( United Nations General Assembly, 2022). Therefore, to achieve health equity for rare diseases, it is necessary to fund or incentivise orphan drug development. Demand-side incentive policies such as reduced patient cost-sharing can increase revenue potential and encourage innovation ( Iizuka & Uchida, 2017), while supply-side incentives such as grants, protocol assistance and administrative and procedural guidance can encourage pharmaceutical companies to pursue orphan designation ( Aartsma-Rus *et al*., 2021; Seoane-Vazquez *et al*., 2008).

Given the incurability of many rare diseases, as well as the lack of treatment plans, most of the costs associated with rare diseases can be attributed to care management or supportive therapies. Cost-of-illness studies can be used to estimate the direct, indirect and associated costs of living with a rare disease for children and their families, as well as the impact of rare diseases on healthcare resources and labour productivity ( Larg & Moss, 2011). Cost-of-illness studies explore the impact of illnesses or diseases on the health outcomes of both individuals and national populations. Overall, the goal of these studies is to evaluate the economic burden of specific illnesses from a societal perspective. Incidence of disease, resultant impacts on morbidity and quality of life, and financial consequences (direct or indirect) due to disability, injury or premature mortality are all approaches that can be explored through cost-of-illness studies ( Jo, 2014). Furthermore, these studies can be conducted prospectively or retrospectively to patients’ healthcare use or treatment, or through a top-down, bottom-up or econometric approach ( Jo, 2014). These approaches address economic impact in different ways: top-down estimates consider the costs of exposure to the disease or the risk factors; bottom-up estimates consider the costs through the number of health inputs used and their unit costs; econometric estimates involve cost comparisons between a cohort with a disease and an unaffected cohort ( Jo, 2014). Cost-of-illness studies are the most common method of assessing the economic burden of rare diseases ( Delaye *et al.*, 2022). Similarly, economic evaluations analyse costs and outcomes of interventions to determine cost effectiveness. There are four types of economic evaluations: cost minimisation analysis; cost utility analysis; cost-benefit analysis and cost consequences analysis ( Goodacre & McCabe, 2002). While cost-of-illness and economic evaluation studies can be useful in determining economic impacts of interventions, the variety of approaches for conducting these studies means that the literature around this topic lacks standardisation.

Direct costs are the healthcare and non-healthcare costs for which the health system, society, family and individuals with the illness are directly accountable for, whereas indirect costs are associated with productivity losses due to morbidity and mortality that indirectly impact the individual, family, society or employer ( Jo, 2014). Economic costs associated with rare diseases arise from both direct healthcare costs, including those of specialised healthcare service provision, direct non-healthcare costs, such as those of care or education services, and indirect costs, such as loss of income at an individual level and loss of productivity on a societal level ( Chung *et al*., 2023; Zurynski *et al*., 2008). Standard units of measurement for health outcomes include quality-adjusted life years (QALYs), which are years of healthy life (or life quality relative to healthy life) lived, and disability-adjusted life years (DALYs), which measure the loss of one year of healthy life due to disability or premature mortality ( Feng *et al*., 2020; Jo, 2014).

Studies have been performed to estimate the burden and health-related quality of life for all patients (including children) with rare diseases, such as the Social Economic Burden and Health-Related Quality of Life in Patients with Rare Diseases in Europe (BURQOL-RD) study, which considered eight EU countries (Bulgaria, France, Germany, Hungary, Italy, Sweden, Spain, UK) ( López-Bastida *et al*., 2016); and the National Economic Burden of Rare Disease study, commissioned in the US by the EveryLife Foundation ( Yang *et al*., 2022). These studies both found a significant economic impact of rare diseases due to their high collective prevalence in their populations and high per-person costs, generated either through loss of labour productivity or the cost of formal or informal care. Children with rare diseases in particular have been associated with higher economic costs; in the US, per-person direct costs are most expensive in childhood and decrease with age; similarly, per-person indirect costs for children are higher than for adults, due to absenteeism, presenteeism and social productivity losses of family caregivers ( Yang *et al*., 2022). In Hong Kong, children with rare diseases were also found to experience higher per-person direct and indirect costs due to experiencing a higher cost of healthcare services, medical resources, informal care support and special education ( Chung *et al*., 2023). Similarly, families of children living with rare diseases in Spain were identified as having poorer financial situations than families of adults with rare diseases, due to the eligibility of adults for benefits such as treatment allowances or pensions, which are not available for children ( Gimenez-Lozano *et al*., 2022). These disproportionately higher per-person costs for children have a significant impact on families. Capturing the evidence on this economic impact can aid policy makers in their decision-making, inform resource allocation and lead to the development of solutions that can benefit people living with rare diseases and their families ( Yang *et al*., 2022).

The economic impact of rare diseases is somewhat neglected within the rare disease literature compared to other areas, e.g., clinical research, likely due to the heterogeneity and low individual incidence of rare diseases. Few cost-of-illness studies specifically related to children have been performed, and those that exist tend to focus on rare diseases for which a specific treatment exists (e.g., cystic fibrosis) ( Angelis *et al.*, 2015). As 95% of rare diseases lack an approved treatment ( Kruse *et al*., 2022; Somanadhan *et al*., 2020), this means that economic evaluations are extremely limited. Previous scoping reviews on these evaluations in general have found a lack of studies on the economic impact of rare diseases ( Delaye *et al.*, 2022; García-Pérez *et al*., 2021). Furthermore, the quality of evidence is often below regulatory standards due to the inability to conduct quality experiments (e.g., randomised control trials) due to small sample size or the difficulty in capturing the patient experience. Therefore, standard economic evaluation procedures may not be fully appropriate for rare diseases ( Armeni *et al*., 2021).

The goal of this scoping review is to map existing studies on the economic impact of living with a rare disease for children and their families, examine the current methodologies for collating and reporting the evidence of these impacts, and to identify any potential gaps in the literature. A preliminary search of MEDLINE and the Cochrane Library was conducted to identify any existing reviews on a similar topic. While there are reviews on the economic impact of rare diseases in all populations ( Delaye *et al.*, 2022; García-Pérez *et al*., 2021); these differ to this proposed review which will focus on a child and family perspective. A scoping review will be conducted, to examine how the cost of rare diseases has been explored to date, the approaches used, and to identify the gaps within this literature; this aligns with the indications for conducting a scoping review as set by the Joanna Briggs Institute (JBI) guidance ( Peters *et al*., 2020a).

## Methods

The proposed systematic scoping review will be conducted in accordance with the updated JBI methodology for scoping reviews ( Peters *et al*., 2020b) and recommendations from Levac *et al.* (2010), which build on previous existing methodological guidance ( Arksey & O’Malley, 2005; Peters *et al*., 2015). A six-stage process, including knowledge user consultation as a final stage, will be employed throughout the review process and has been used in the development of this scoping review protocol. An overview of each stage is presented below. Section headings are taken from Levac *et al.* (2010).

Reporting will be guided by the Preferred Reporting Items for Systematic Reviews and Meta Analyses Scoping Review extension (PRISMA-ScR) checklist, to ensure compliance with up-to-date PRISMA reporting guidance. This extension was developed to improve the methodological and quality reporting of scoping reviews ( Tricco *et al*., 2018), and will assist in the formulation of the review and an informal quality assessment.

A panel of patient and public involvement (PPI) representatives from the rare disease community and knowledge users working in the rare disease field will be invited to validate the results and their presentation, according to the guidance on knowledge user engagement developed by Pollock *et al*. (2022). The review will be conducted as part of a doctoral project that was developed through public-patient involvement. The topic and a relationship with the knowledge users and PPI representatives were established prior to the commencement of this review via the Rare Disease Research Partnership (RAinDRoP) ( Somanadhan *et al*., 2020). This protocol has been developed with and reviewed by knowledge users and PPI representatives to inform the research questions and search strategy of the review, as per the knowledge user engagement guidance ( Pollock *et al*., 2022).

### Stage 1: Identifying the research question

***Research question***

This scoping review aims to identify, appraise and reliably map current literature on the economic impacts of living with rare diseases for children and their families, and identify the screening and assessment tools used to identify these impacts. Therefore, the research question is “what is the economic impact for families and caregivers of children with a rare disease, and how are these impacts identified?”.

The research objectives of this scoping review are:

1. To identify, appraise, and synthesise knowledge surrounding the economic impacts of rare diseases affecting children and their families.
2. To understand how these impacts are measured.
3. To clarify what specific disease populations and disease characteristics are most researched.
4. To determine the study settings, rare conditions and geographical contexts, and the study types and organizations involved (e.g., charitable organisations, pharmaceutical companies, etc.).
5. To highlight any gaps in the literature on the economic impact of rare diseases on children and their families.

As scoping reviews summarise particular topics, the research questions are typically broad with a specific scope of inquiry, indicating the focus of the study ( Levac *et al.*, 2010). Therefore, the following sections explain the Population-Concept-Context (PCC) framework ( Peters *et al*., 2020a) used to formulate the research questions for this study, to illustrate the origins of the research questions and the search strategy.

***PCC Framework***

*Population*

This scoping review focuses on the economic impact of having a child with a rare disease on family units, recognising that children will not bear the economic burden of living with a rare disease themselves. Therefore, studies that consider the economic impact from the perspective of families, parents, (informal) caregivers that are part of the family unit or guardians will be included. This includes the direct and indirect impacts of having a child with a rare disease which commonly affect family members.

*Concept*

The concept is the economic impact of rare diseases for children and/or their families. This is assumed to mean any financial experiences or effects directly or indirectly incurred due to the rare disease. How these experiences/ effects are measured (e.g., using validated screening or measurement tools) will also be mapped. The economic impact of rare diseases is typically assessed through cost-of-illness studies or economic evaluations, as these studies explore the impact of illnesses or diseases via their associated costs ( García-Pérez *et al*., 2021). Therefore, these types of studies are highlighted in the search strategy. Other studies, e.g., intervention or health impact studies that discuss economic burden or impact for families, are also captured in the search strategy.

*Context*

The context differs to the population, as this scoping review will focus on the phenomenon of children with rare diseases. Therefore, the context is rare diseases in childhood, and is thus divided into two parts. The United Nations Convention on the Rights of the Child definition is used to understand a ‘child’ as anyone under the age of 18 years ( UNICEF, n.d.). Other relevant terms, such as adolescent, infant and variations on these are included to capture relevant literature. Studies that discuss siblings will also be included, to reflect the impact of living with a rare disease on this population.

The US and European definitions of rare diseases will be adhered to, dependent on the context of the articles. Studies that consider individual rare diseases will be included; however, studies that consider chronic or complex conditions that are not rare will be excluded. Studies that discuss a range of complex conditions, including rare disease(s), will be included if they clearly delineate their results and data can be charted according to the rare disease(s) considered. Due to variation in definitions, illnesses or diseases that are conceptualised as rare diseases by researchers in particular countries where these diseases are considered rare will be included, even if different to the authors’ home country. For example, sickle cell disease is considered rare in the Republic of Ireland but is the most common genetic condition in the world ( Rare Disease Taskforce, 2020).

***Types of sources***

All study designs, whether quantitative and qualitative, will be considered for the review. This will include both experimental and quasi-experimental study designs including randomised controlled trials, non-randomised controlled trials, before and after studies and interrupted time-series studies. Analytical observational studies including prospective and retrospective cohort studies, case-control studies and analytical cross-sectional studies will also be considered for inclusion, as will descriptive observational study designs including case series, individual case reports and descriptive cross-sectional studies.

Qualitative studies will also be considered including, but not limited to, designs such as phenomenology, grounded theory, ethnography, qualitative description, action research and feminist research.

Studies that are not primary research, e.g., reviews or conference materials, or non-peer-reviewed articles, e.g., dissertations or theses, will be excluded, as will studies that have not been peer-reviewed. A publication date limit of 1983 until the present will be applied to ensure relevance to current economic contexts. The start date of 1983 reflects the introduction of the United States Orphan Drug Act in the same year, which aimed to advance development of drug treatments for rare diseases and thus stimulated research in this area ( Dawkins *et al*., 2018). There are no limits on geographical location. Articles in languages other than English will be excluded due to lack of funding for translation services; however, this restriction will not be imposed on the search strategy, and it may be reviewed if there are a small number of results in another language, to reduce the possibility of language bias ( Stern & Kleijnen, 2020). Results excluded due to language will be reported transparently. The corresponding authors of articles for which the full-text is not available online will be contacted to source a copy of the article; however, if it is not possible to retrieve the full-text, the article will be excluded. The inclusion and exclusion criteria are presented in Table 1.

**Table 1.** Inclusion and exclusion criteria.

	Inclusion	Exclusion
**Population**	Studies that discuss costs of rare diseases to the family unit, including parents, informal caregivers and/ or guardians.	Studies that only focus on populations outside the family unit, e.g., caregivers or guardians not related to the child with a rare disease (e.g., formal/ professional caregivers), healthcare providers, healthcare systems, society.
**Concept**	Studies that discuss the economic impacts of having a rare disease on children and their families, including direct and indirect impacts.	Studies that discuss the impacts of rare diseases on children and their families, but do not focus on economic impacts.
**Context**	Any geographical location. Studies that focus on rare diseases in childhood.	Studies that do not differentiate their study population between children or adults. Studies that discuss complex or chronic conditions that are not rare diseases.
**Language**	Articles in English.	Articles in languages other than English.
**Publication Date**	1983 to present.	Articles published prior to 1983.
**Types of Evidence ** **Sources**	Peer-reviewed cost-of-illness studies, economic evaluations or other studies that discuss the economic impacts of living with a rare disease for children and their families. Peer-reviewed primary research sources e.g., research studies.	Secondary research sources, e.g., reviews (systematic, scoping, meta-analyses, etc.). Conference materials, editorials, commentaries, opinion papers, dissertations/theses. Studies for which the full text is not available following contact with the corresponding author. Non-peer-reviewed research.

### Stage 2: Identifying relevant studies

Databases to be included in this scoping review were chosen to reflect the nature of the research questions. Therefore, the databases searched will include EconLit (EBSCO), ABI/Inform Global (Proquest), MEDLINE (Ovid), PubMed, CINAHL Plus, and Scopus (Elsevier).

The search strategy was developed by the primary author in consultation with a university librarian. An initial search was performed on 13 March 2023 to establish common terms and subject headings relevant to the topic. These terms and headings were used to develop the search strategy. Boolean operators, and truncation markers will be used in the search strategy, which will be altered to meet the requirements of each database. A sample search strategy is provided in Table 2. This search strategy is subject to change during the review process, as the reviewers become more familiar with the literature ( Peters *et al*., 2020a).

**Table 2.** Sample search strategy.

Search	Search Terms
**1**	((famil*) OR (parent*) OR (caregiver*) OR (guardian*))
**2**	((cost of illness) OR (economic evaluation*) OR (economic burden) OR (burden of illness) OR (economic cost*) OR (economic impact*) OR (financial hardship))
**3**	((paediatric) OR (child*) OR (adolescen*) OR (sibling*) OR (newborn*) OR (infan*))
**4**	((rare disease*) OR (rare condition*) OR (orphan disease*) OR (orphan condition*) OR (ultra-rare disease*) OR (ultra-rare condition*) OR (ultra-orphan disease*) OR (ultra-orphan condition*))
**5**	S3 AND S4
**6**	S1 AND S2 AND S5

### Stage 3: Study selection

Studies yielded by the search strategy will be uploaded to EndNote 20 ( Clarivate Analytics, 2023) and duplicates will be removed. The remaining articles will be screened by title and abstract first, followed by a full-text review to determine their suitability for inclusion in the review. This part of the proposed review will be supported through the use of Covidence, a web-based collaboration software platform that streamlines the production of systematic and other literature reviews ( Covidence, n.d.). Screening will be performed by the primary author and another independent reviewer and validated through peer debriefing. Disagreements on whether to include or exclude articles will be addressed by a third researcher. A pilot test will be completed with a sample of studies (n=25) to ensure consistent decision-making between the reviewers and the clarity of the inclusion and exclusion criteria, and facilitate possible necessary refinements, as recommended by the JBI Manual for Evidence Synthesis ( Peters *et al*., 2020a).

Following title and abstract screening, included articles will go through a full-text review to determine their inclusion eligibility according to the inclusion and exclusion criteria. As in the previous study selection step, this will be conducted by the primary author and another independent reviewer and validated through peer debriefing, with any disagreements resolved by a third researcher. Reasons for exclusion of sources of evidence at full text that do not meet the inclusion criteria will be recorded and reported in the scoping review. As discussed in the previous section, authors of papers will be contacted to request missing or additional data, where required. The reference lists of included articles will also be searched to identify other potential studies not captured by the search strategy, and these will undergo a similar full text screening process by two researchers.

**Figure 1.**
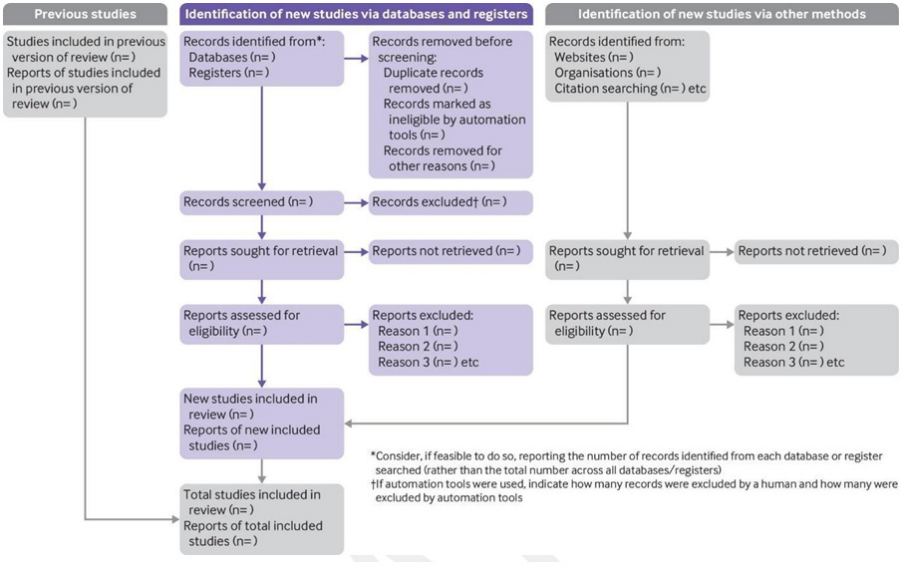
PRISMA 2020 Flow Diagram Template for Systematic Reviews, taken from Page *et al.* (2021).

The actions of screening and full-text review will be reported according to the PRISMA-ScR guidance ( Tricco *et al*., 2018). A Preferred Reporting Items for Systematic reviews and Meta-Analyses (PRISMA) 2020 flow diagram template ( Figure 1) for systematic reviews ( Page *et al*., 2021) will be used to illustrate the screening process for transparency and replicability. The JBI Template source of evidence details, characteristics and results extraction instrument ( Peters *et al*., 2020a) will be amended to the concept of this scoping review and used to describe the included articles for transparency.

A quality appraisal will be performed on included articles to determine the trustworthiness of the results. While not an essential step in scoping reviews ( Arksey & O’Malley, 2005; Levac *et al.*, 2010), this step will be performed due to the evidence of quality concerns within the field of economic evaluations of rare diseases, as discussed in a previous section. The Mixed Methods Appraisal Tool (MMAT) ( Hong *et al*., 2018) will be used due to its versatility for appraising quantitative, qualitative or mixed methods studies. The results of the quality appraisal will be reported in the final scoping review.

### Stage 4: Charting the data

The data extracted will include specific details about the participants, concept, context, study methods and key findings relevant to the review questions. A draft data charting extraction table developed ( Table 3) will be trialled at the pilot test stage and will be modified and revised as necessary both prior and during the process of extracting data from each included evidence source. This draft data extraction table has been developed using guidance from the JBI Manual for Evidence Synthesis ( Peters *et al*., 2020a) and amended to the focus of the proposed scoping review. Modifications will be detailed in the scoping review. Any disagreements that arise between the reviewers will be resolved through discussion, or with an additional reviewer/s. If appropriate, authors of papers will be contacted to request missing or additional data, where required.

**Table 3.** Sample data charting table.

Heading	Description of Data
Author(s)	The authors responsible for the source.
Year of Publication	When the source was published.
Origin/ Country of Origin	Where the source was published or conducted.
Aims	What the source set out to do.
Sample	The sample used within the source, including characteristics e.g., size, age, gender, etc.
Methodology	The research design and procedures or techniques used to identify, select, process, and analyse the data obtained by the authors.
Economic Impacts	What economic impacts were discussed by the source, and how these were measured.
Interventions	Where the source discusses the introduction of an intervention, what type of intervention it was, its comparator and the details of its introduction.
Other Key Findings	Any other key points related to the scoping review research questions.

Knowledge users and PPI representatives will be provided the opportunity to participate in Stage 3 and Stage 4 of the scoping review process. If interested in participating, they will receive training on the software used for data screening and extraction.

### Stage 5: Collating, summarising and reporting the results

Data charting will be completed and collated on Microsoft Excel and will be shared among the team for review and sign off. The results of the review will be presented as a map of the data from the included studies according to the aims of the review, as recommended by the JBI Manual for Evidence Synthesis ( Peters *et al*., 2020a). This will involve two aspects: reporting of the overall results, which will be presented in tabular form and include aspects such as number of studies included, types of study design, etc; and a basic descriptive analysis, which will apply meaning to the results ( Levac *et al.*, 2010). Thematic analysis will not be undertaken as it is outside the remit of scoping reviews ( Peters *et al*., 2020a); however, a table according to the PAGER framework will be provided. This framework, which looks at the patterns (P), advances (A), gaps (G), Evidence for practice (E) and research recommendations (R), provides a consistent approach for the reporting of scoping review findings ( Bradbury-Jones *et al*., 2022). Explicit details of the methodology for analysis will be provided to ensure transparency and rigour in the analysis process. The results of the review will be validated through peer debriefing and PPI representative and knowledge user consultation, discussed in the next section. Gaps in the literature will be highlighted as areas for future research.

### Stage 6: Consultation

The results of the review will be presented to PPI representatives and knowledge users working in the rare disease field for validation and to ensure the implications of the findings are meaningful to their community. For the purposes of this review, a knowledge user is defined as:

*“one in a position of authority to influence and/or make decisions about health policy or the delivery of services and can act to ensure that the findings of the research will be translated to influence decision making and change within their (or other) organisations” ( Health Research Board, 2023, p.7).*

Dissemination of the research findings will also be developed through knowledge user engagement. The same knowledge users and PPI representatives will be invited to participate in the development of an evidence summary for dissemination on social media. This is to ensure knowledge translation and impact is accessible and meaningful to the communities it is targeted at and is in line with the guidance for knowledge user engagement developed by Pollock *et al*. (2022).

As the PRISMA-ScR tool currently does not include the reporting of knowledge user engagement ( Pollock *et al*., 2022), the participation of knowledge users in the consultation process will be reported using the ACTIVE framework, developed by Pollock *et al*. (2019) for use in systematic reviews. This framework is appropriate for use in this review due to the similarities in conduct processes between systematic and scoping reviews ( Pollock *et al*., 2022).

Knowledge users in clinical practice and policy, and PPI representatives have been consulted in the development of this scoping review protocol and have validated the research questions proposed. These knowledge users and PPI representatives will be involved in the review process through consultation and review.

## Dissemination

The proposed scoping review will be submitted for publication in peer-reviewed academic journals. As previously discussed, an evidence summary will be developed for social media through knowledge user engagement, to ensure the meaningful transmission of results. In addition, the results will be presented at conferences and shared with relevant stakeholders and policymakers where appropriate.

## Study Status

This review is currently in the stage three: study selection phase. Database searches have been performed and the results are being screened by title and abstract by two reviewers.

## Conclusions

Rare diseases are associated with significant economic impacts for families of children living with rare diseases; however, the literature in this area is sparse and as a topic, it remains underexplored. The results of this scoping review will map existing work on the economic impacts of living with a rare disease for children and their families, contributing to the literature on this topic. The results will also be used to develop a survey measuring the economic and psychosocial costs of living with a rare disease in Northern Ireland and the Republic of Ireland, and to develop a cost-minimal intervention prototype around these economic impacts in the future, as part of this doctoral project.

## Data Availability

No data are associated with this article.
