# The economic impact of living with a rare disease for children and their families: a scoping review protocol

**DOI:** 10.12688/hrbopenres.13765.2

**Published:** 2024-04-08

**Authors:** Niamh Buckle, Orla Doyle, Naonori Kodate, Suja Somanadhan

**Affiliations:** 1 School of Nursing, Midwifery and Health Systems, University College Dublin, Dublin, Leinster, D04 V1W8, Ireland; 2 School of Economics, University College Dublin, Dublin, Leinster, D04 N9Y1, Ireland; 3 School of Social Policy, Social Work and Social Justice, University College Dublin, Dublin, Leinster, D04 N9Y1, Ireland; 4 UCD Centre for Interdisciplinary Research, Education and Innovation in Health Systems (UCD IRIS), University College Dublin, Dublin, Leinster, Ireland

**Keywords:** Child, cost-of-illness, economic evaluation, family, financial hardship, orphan diseases, rare diseases, ultra-rare diseases

## Abstract

**Background:**

Rare diseases are an often chronic, progressive and life-limiting group of conditions affecting more than 30 million people in Europe. These diseases are associated with significant direct and indirect costs to a spectrum of stakeholders, ranging from individuals and their families to society overall. Further quantitative research on the economic cost for children and their families living with a rare disease is required as there is little known on this topic. This scoping review aims to document the extent and type of evidence on the economic impacts of living with a rare disease for children and their families.

**Methods:**

This scoping review will follow the PRISMA-ScR and Joanna Briggs Institute guidelines and follow the six-stage methodology for scoping reviews: (1) identifying the research question, (2) identifying relevant studies, (3) study selection, (4) charting the data, (5) collating, summarising and reporting results and (6) knowledge user consultation. Key inclusion criteria have been developed according to the Population-Concept-Context (PCC) framework. The databases EconLit, ABI/Inform, MEDLINE, PubMed, CINAHL, and Scopus will be searched for possible articles for inclusion. Two independent reviewers will screen titles and abstracts of potential articles using a dual review process to ensure all relevant studies are included. All included articles will be assessed using a validated quality appraisal tool. A panel of patient and public involvement representatives experiencing rare diseases and knowledge users will validate the review results.

**Conclusions:**

This scoping review will map the current literature on the economic impact of paediatric rare diseases to understand how these impacts affect children living with rare diseases and their families. This evidence has the potential to influence policy and future research in this area and will support further research on the economic impact of rare diseases on families.

## Introduction

The collective incidence of rare diseases contributes to a significant global population that belies its ‘rare’ title. It is estimated that there are between 6,000 and 8,000 known rare diseases ( European Commission, n.d.; Kruse *et al*., 2022) that affect 3.5%-5.9% of the global population ( Nguengang Wakap *et al*., 2020). Rare diseases are often incurable, complex and are associated with a high social and economic burden that reaches from the patient to society ( García-Pérez *et al*., 2021; Somanadhan *et al*., 2023a). Approximately 70% of rare diseases start in childhood, and early diagnosis and treatment is critical to prevent disease progression and avoid adverse health outcomes, including disability and death ( Morton *et al*., 2022).

Eighty percent of rare diseases have a genetic component and therefore treatment is often limited or costly, and 95% of rare diseases lack an approved treatment ( Somanadhan *et al*., 2023b). This brings challenges regarding funding for research and development and treatment accessibility, as orphan drugs, which are developed specifically for rare diseases, can cost up to five times more than non-orphan drugs ( Chambers *et al*., 2020). Few rare diseases have orphan drugs, contributing to a large unmet need ( Rodriguez-Monguio *et al.*, 2017). Other treatments for rare diseases, such as gene therapy, which involves modification of genes to treat, prevent or cure an illness or disease ( White, 2019), similarly come at a high cost and are limited.

The lack of funding for orphan drug development stems from the limited market potential of rare diseases, as developing drugs for limited patient groups is unlikely to be cost effective for pharmaceutical companies ( Iizuka & Uchida, 2017). However, this has both human and economic costs. High prices and reduced access to treatment can cause increased health burden and premature death for patients with rare diseases; lack of treatment access also reduces rare disease patients’ capacity to engage in society independently, incurring costs such as loss of income tax, absenteeism and presenteeism of carers, and potential increased healthcare costs due to the consequences of poor health ( Hyry *et al*., 2013).

Given the incurability of many rare diseases, as well as the lack of treatment plans, most of the costs associated with rare diseases can be attributed to care management or supportive therapies. Cost-of-illness studies can be used to estimate the direct, indirect and associated costs of living with a rare disease, as well as the impact of rare diseases on healthcare resources and labour productivity ( Larg & Moss, 2011). Other methods of determining economic burden are economic evaluations, which analyse costs and outcomes of interventions to determine cost-effectiveness ( Goodacre & McCabe, 2002). Direct costs are the healthcare and non-healthcare costs for which the health system, society, family and individuals with the illness are directly accountable for, whereas indirect costs are associated with productivity losses due to morbidity and mortality that indirectly impact the individual, family, society or employer ( Jo, 2014). Economic costs associated with rare diseases arise from both direct healthcare costs, including those of specialised healthcare service provision, direct non-healthcare costs, such as those of care or education services, and indirect costs, such as loss of income at an individual level and loss of productivity on a societal level ( Chung *et al*., 2023; Zurynski *et al*., 2008). Incidence of disease, resultant impacts on morbidity and quality of life, and financial consequences (direct or indirect) due to disability, injury or premature mortality are all approaches that can be explored through cost-of-illness studies ( Jo, 2014). While cost-of-illness and economic evaluation studies can be useful in determining economic impacts of interventions, the variety of approaches for conducting these studies means that the literature around this topic lacks standardisation.

Studies have been performed to estimate the burden and health-related quality of life for all patients (including children) with rare diseases, such as the Social Economic Burden and Health-Related Quality of Life in Patients with Rare Diseases in Europe (BURQOL-RD) study, which considered eight EU countries (Bulgaria, France, Germany, Hungary, Italy, Sweden, Spain, UK) ( López-Bastida *et al*., 2016); and the National Economic Burden of Rare Disease study, commissioned in the US by the EveryLife Foundation ( Yang *et al*., 2022). These studies both found a significant economic impact of rare diseases due to their high collective prevalence and high per-person costs, generated either through loss of labour productivity or the cost of formal or informal care. Children with rare diseases have been associated with higher economic costs; in the US, per-person direct costs are most expensive in childhood and decrease with age; similarly, per-person indirect costs for children are higher than for adults, due to absenteeism, presenteeism and social productivity losses of family caregivers ( Yang *et al*., 2022). In Hong Kong, children with rare diseases were also found to experience higher per-person direct and indirect costs due to experiencing a higher cost of healthcare services, medical resources, informal care support and special education ( Chung *et al*., 2023). Similarly, families of children living with rare diseases in Spain were identified as having poorer financial situations than families of adults with rare diseases, due to the eligibility of adults for benefits such as treatment allowances or pensions, which are not available for children ( Gimenez-Lozano *et al*., 2022). These disproportionately higher per-person costs for children have a significant impact on families. Capturing the evidence on this economic impact can aid policy makers in their decision-making, inform resource allocation and lead to the development of solutions that can benefit people living with rare diseases and their families ( Yang *et al*., 2022). However, the economic impact of rare diseases is somewhat neglected within the rare disease literature compared to other areas, e.g., clinical research, likely due to the heterogeneity and low individual incidence of rare diseases. Apart from the studies discussed above, few cost-of-illness studies specifically related to children have been performed, and those that exist tend to focus on rare diseases for which a specific treatment exists (e.g., cystic fibrosis) ( Angelis *et al.*, 2015). Previous scoping reviews in general have found a lack of studies on the economic impact of rare diseases ( Delaye *et al.*, 2022; García-Pérez *et al*., 2021).

The goal of this scoping review is to map existing studies on the economic impact of living with a rare disease for children and their families, examine the current methodologies for collating and reporting the evidence of these impacts, and to identify any potential gaps in the literature. It examines the types of direct and indirect impacts that are evaluated in the literature, and how they are measured and analysed. This study does not conduct a meta-analysis to determine the average monetary impact of rare diseases found across studies; rather, this review focuses on a family economic perspective, considering rare diseases amongst paediatric populations. To the author’s knowledge, it is the first scoping review with this focus.

## Methods

The proposed systematic scoping review will be conducted in accordance with the updated JBI methodology for scoping reviews ( Peters *et al*., 2020b) and recommendations from Levac *et al.* (2010), which build on previous existing methodological guidance ( Arksey & O’Malley, 2005; Peters *et al*., 2015). A six-stage process, including knowledge user consultation as a final stage, will be employed throughout the review process and has been used in the development of this scoping review protocol. An overview of each stage is presented below. Section headings are taken from Levac *et al.* (2010).

Reporting will be guided by the Preferred Reporting Items for Systematic Reviews and Meta Analyses Scoping Review extension (PRISMA-ScR) checklist, to ensure compliance with up-to-date PRISMA reporting guidance. This extension was developed to improve the methodological and quality reporting of scoping reviews ( Tricco *et al*., 2018), and will assist in the formulation of the review and an informal quality assessment.

The review will be conducted as part of a wider project with input from four patient and public involvement (PPI) representatives from the rare disease community, and six knowledge users working in the rare disease field already linked with the project will validate the results and their presentation. This is according to the guidance on knowledge user engagement developed by Pollock *et al*. (2022). Relationships with the knowledge users and PPI representatives were established prior to the commencement of this review via the Rare Disease Research Partnership (RAinDRoP) ( Somanadhan *et al*., 2020). This protocol has been developed with and reviewed by knowledge users and PPI representatives to inform the research questions and search strategy, as per the knowledge user engagement guidance ( Pollock *et al*., 2022). Further information is provided in the relevant section of this protocol (Stage 6: Consultation).

### Stage 1: Identifying the research question

***Research question***

This scoping review aims to identify, appraise and reliably map current literature on the economic impacts of living with rare diseases for children and their families, and identify the screening and assessment tools used to identify these impacts. Therefore, the research question is “how is the economic impact (direct and indirect costs) for families and caregivers of children with a rare disease identified and measured in the literature?”.

The research objectives of this scoping review are:

1. To identify, appraise, and synthesise knowledge surrounding the economic impacts of rare diseases affecting children and their families through consideration of healthcare and non-healthcare direct and indirect costs.
2. To understand how these costs are measured.
3. To clarify what specific disease populations and disease characteristics are most researched.
4. To determine the study settings, rare conditions and geographical contexts, and the study types and organizations involved (e.g., charitable organisations, pharmaceutical companies, etc.).
5. To highlight any gaps in the literature on the economic impact of rare diseases on children and their families.

As scoping reviews summarise particular topics, the research questions are typically broad with a specific scope of inquiry, indicating the focus of the study ( Levac *et al.*, 2010). Therefore, the following sections explain the Population-Concept-Context (PCC) framework ( Peters *et al*., 2020a) used to formulate the research questions for this study, to illustrate the origins of the research questions and the search strategy.

***PCC Framework***

*Population*

This scoping review focuses on the economic impact of having a child with a rare disease on family units, recognising that children will not bear the economic burden of living with a rare disease themselves. Therefore, studies that consider the economic impact from the perspective of families, parents, (informal) caregivers that are part of the family unit or guardians will be included. This includes the direct and indirect impacts of having a child with a rare disease which commonly affect family members.

*Concept*

The concept is the economic impact of rare diseases for children and/or their families. This is assumed to mean any financial costs directly or indirectly incurred due to the rare disease. The types of direct ad indirect costs that will be included in this review have been identified ( Delaye *et al*., 2022; Fautrel *et al*., 2020; Liljas, 1998) and are included in Table 1.

**Table 1.** Types of Direct and Indirect Costs.

DIRECT COSTS		INDIRECT COSTS
Medical	Non-Medical	
Inpatient care	Care (professional)	Absenteeism (missed workdays)
Outpatient care	Education-related costs	Forced/ early retirement
GP visits	Respite care	Informal care
Other healthcare professional visits (e.g., physiotherapist, occupational therapist/ etc.)	Alternative and complementary medicine	Housing adaptations
Medication	Transportation/ Travel costs	Costs accrued by family and/ or friends for caring duties
Medical equipment and/ or assistive devices	Insurance costs	Presenteeism (loss of productivity due to illness or other conditions)
Other out-of-pocket costs		
*Delaye *et al*. (2022); Fautrel *et al.* (2020); Liljas (1998)*		

How these effects are measured (e.g., using validated screening or measurement tools) will also be mapped. The economic impact of rare diseases is typically assessed through cost-of-illness studies or economic evaluations, as these studies explore the impact of illnesses or diseases via their associated costs ( García-Pérez *et al*., 2021). Therefore, these types of studies are highlighted in the search strategy. Other studies, e.g., intervention or health impact studies that discuss economic burden or impact for families, are also captured in the search strategy.

*Context*

The context differs to the population, as this scoping review will focus on the phenomenon of children with rare diseases. Therefore, the context is rare diseases in childhood, and is thus divided into two parts. The first centres around the definition of ‘children’. The United Nations Convention on the Rights of the Child definition is used to understand a ‘child’ as anyone under the age of 18 years ( UNICEF, n.d.). Other relevant terms, such as adolescent, infant and variations on these are included to capture relevant literature. Studies that discuss siblings will also be included, to reflect the impact of living with a rare disease on this population.

The second part of the context of this review considers rare diseases. For the purposes of this review, conditions that are classified as rare by Orphanet will be considered rare diseases. This inventory is cross-referenced with international terminologies and reference databases ( Orphanet, 2020) and should therefore help the classification of conditions between the included studies, or in studies that include multiple contexts. Studies that consider individual rare diseases will be included; however, studies that consider chronic or complex conditions that are not rare will be excluded. Studies that discuss a range of complex conditions, including rare disease(s), will be included if they clearly delineate their results and data can be charted according to the rare disease(s) considered. Due to variation in definitions, illnesses or diseases that are conceptualised as rare diseases by researchers in particular countries where these diseases are considered rare will be included, even if different to the authors’ home country. For example, sickle cell disease is considered rare in the Republic of Ireland but is the most common genetic condition in the world ( Rare Disease Taskforce, 2020).

***Types of sources***

All study designs, whether quantitative and qualitative, will be considered. Studies of any design that provide information on the economic costs (direct and/ or indirect) of paediatric rare diseases and meet the inclusion criteria will be included. This will include both experimental and quasi-experimental study designs such as randomised controlled trials, non-randomised controlled trials, before and after studies and interrupted time-series studies. Analytical observational studies including prospective and retrospective cohort studies, case-control studies and analytical cross-sectional studies will also be considered for inclusion, as will descriptive observational study designs including case series, individual case reports and descriptive cross-sectional studies.

Qualitative studies will also be considered including, but not limited to, designs such as phenomenology, grounded theory, ethnography, qualitative description, action research and feminist research.

Studies that are not primary research, e.g., reviews or conference materials, or non-peer-reviewed articles, e.g., dissertations or theses, will be excluded, as will studies that have not been peer-reviewed. A publication date limit of 1983 until the present will be applied to ensure relevance to current economic contexts. The start date of 1983 reflects the introduction of the United States Orphan Drug Act in the same year, which aimed to advance development of drug treatments for rare diseases and thus stimulated research in this area ( Dawkins *et al*., 2018). There are no limits on geographical location. Articles in languages other than English will be excluded due to lack of funding for translation services; however, this restriction will not be imposed on the search strategy, and it may be reviewed if there are a small number of results in another language, to reduce the possibility of language bias ( Stern & Kleijnen, 2020). If results are excluded due to language, the reasoning for this will be reported. The corresponding authors of articles for which the full-text is not available online will be contacted to source a copy of the article; however, if it is not possible to retrieve the full-text, the article will be excluded. The inclusion/exclusion criteria are presented in Table 2.

**Table 2.** Inclusion and exclusion criteria.

	Inclusion	Exclusion
**Population**	Studies that discuss costs of rare diseases to the family unit, including parents, informal caregivers and/ or guardians.	Studies that only focus on populations outside the family unit, e.g., caregivers or guardians not related to the child with a rare disease (e.g., formal/ professional caregivers), healthcare providers, healthcare systems, society.
**Concept**	Studies that discuss the economic impacts of having a rare disease on children and their families, including direct and indirect impacts.	Studies that discuss the impacts of rare diseases on children and their families, but do not focus on economic impacts.
**Context**	Any geographical location. Studies that focus on rare diseases in childhood.	Studies that do not differentiate their study population between children or adults. Studies that discuss complex or chronic conditions that are not rare diseases.
**Language**	Articles in English.	Articles in languages other than English.
**Publication Date**	1983 to present.	Articles published prior to 1983.
**Types of Evidence ** **Sources**	Peer-reviewed cost-of-illness studies, economic evaluations or other studies that discuss the economic impacts of living with a rare disease for children and their families. Peer-reviewed primary research sources e.g., research studies.	Secondary research sources, e.g., reviews (systematic, scoping, meta-analyses, etc.). Conference materials, editorials, commentaries, opinion papers, dissertations/theses. Studies for which the full text is not available following contact with the corresponding author. Non-peer-reviewed research.

### Stage 2: Identifying relevant studies

Databases to be included in this scoping review were chosen to reflect the nature of the research questions. Therefore, the databases searched will include EconLit (EBSCO), ABI/Inform Global (Proquest), MEDLINE (Ovid), PubMed, CINAHL Plus, and Scopus (Elsevier).

The search strategy was developed by the primary author in consultation with a university librarian. An initial search was performed on 13 March 2023 to establish common terms and subject headings relevant to the topic. These terms and headings were used to develop the search strategy. Boolean operators, and truncation markers will be used in the full search strategy, which will be altered to meet the requirements of each database. A sample search strategy is provided in Table 3. This search strategy is subject to change during the review process, as the reviewers become more familiar with the literature ( Peters *et al*., 2020a).

**Table 3.** Sample search strategy.

Search	Search Terms
**1**	((famil*) OR (parent*) OR (caregiver*) OR (guardian*))
**2**	((cost of illness) OR (economic evaluation*) OR (economic burden) OR (burden of illness) OR (economic cost*) OR (economic impact*) OR (financial hardship))
**3**	((paediatric) OR (child*) OR (adolescen*) OR (sibling*) OR (newborn*) OR (infan*))
**4**	((rare disease*) OR (rare condition*) OR (orphan disease*) OR (orphan condition*) OR (ultra-rare disease*) OR (ultra-rare condition*) OR (ultra-orphan disease*) OR (ultra-orphan condition*))
**5**	S3 AND S4
**6**	S1 AND S2 AND S5

### Stage 3: Study selection

Studies yielded by the search strategy will be uploaded to EndNote 20 ( Clarivate Analytics, 2023) and duplicates will be removed. The remaining articles will be screened by title and abstract first, followed by a full-text review to determine their suitability for inclusion in the review. This part of the proposed review will be supported through the use of Covidence, a web-based collaboration software platform that streamlines the production of systematic and other literature reviews ( Covidence, n.d.). Screening will be performed by the primary author and another independent reviewer and validated through peer debriefing. Disagreements on whether to include or exclude articles will be addressed by a third researcher. A pilot test will be completed with a sample of studies (n=25) to ensure consistent decision-making between the reviewers and the clarity of the inclusion and exclusion criteria, and facilitate possible necessary refinements, as recommended by the JBI Manual for Evidence Synthesis ( Peters *et al*., 2020a).

Following title and abstract screening, included articles will go through a full-text review to determine their inclusion eligibility according to the inclusion/ exclusion criteria. As in the previous study selection step, this will be conducted by the primary author and another independent reviewer and validated through peer debriefing, with any disagreements resolved by a third researcher. Reasons for exclusion of sources of evidence at full text that do not meet the inclusion criteria will be recorded and reported in the scoping review. As discussed in the previous section, authors of papers will be contacted to request missing or additional data, where required. The reference lists of included articles will also be searched to identify other potential studies not captured by the search strategy, and these will undergo a similar full text screening process by two researchers.

The actions of screening and full-text review will be reported according to the PRISMA-ScR guidance ( Tricco *et al*., 2018). A Preferred Reporting Items for Systematic reviews and Meta-Analyses (PRISMA) 2020 flow diagram template for systematic reviews ( Page *et al*., 2021) will be used to illustrate the screening process for transparency and replicability. The JBI Template source of evidence details, characteristics and results extraction instrument ( Peters *et al*., 2020a) will be amended to the concept of this scoping review and used to describe the included articles for transparency.

A quality appraisal will be performed on included articles to determine the trustworthiness of the results. While not an essential step in scoping reviews ( Arksey & O’Malley, 2005; Levac *et al.*, 2010), this step will be performed due to the evidence of quality concerns within the field of economic evaluations of rare diseases. The Consolidated Health Economic Evaluation Reporting Standards 2022 ( Husereau *et al.*, 2022) will be used due to its focus on the methodological quality of economic evaluations. The results of the quality appraisal will be reported in the final scoping review.

### Stage 4: Charting the data

The data extracted will include specific details about the participants, concept, context, study methods and key findings relevant to the review questions. A draft data charting extraction table developed ( Table 4) will be trialled at the pilot test stage and will be modified and revised as necessary both prior and during the process of extracting data from each included evidence source. This table has been developed using guidance from the JBI Manual for Evidence Synthesis ( Peters *et al*., 2020a) and amended to the focus of the proposed scoping review. Modifications will be detailed in the scoping review. The data extraction will be completed by the first author (NB) and audited by the last author (SS). Any disagreements that arise between the reviewers will be resolved through discussion, or with an additional reviewer/s.

**Table 4.** Sample data charting table.

HEADING	DESCRIPTION OF DATA
**General Information**	
Title	Title of paper / abstract / report that data are extracted from.
Lead author contact details	Details of the lead or corresponding author in case contact is required for additional data or clarification.
Rare Disease	What type of Rare Disease is discussed.
Country	Country in which the study was conducted.
**Characteristics of Included Studies**	
Aim of study	What the source set out to do.
Description of Health System	The type and description of the health system relevant to the source.
Study Design	Randomised controlled trial • Non-randomised experimental study • Cohort study • Cross sectional study • Case control study • Systematic review • Qualitative research • Prevalence study • Case series • Case report • Diagnostic test accuracy study • Clinical prediction rule • Economic evaluation • Text and opinion • Other
Methodology	The research design and procedures or techniques used to identify, select, process, and analyse the data obtained by the authors.
**Participants**	
Population Description	The sample used within the source, including characteristics e.g., size, age, gender, etc.
Inclusion Criteria	Inclusion criteria for participation in the study
Exclusion Criteria	Criteria that excluded potential participants from participating in the study
Total number of participants	How many participants were involved in the study.
Baseline population characteristics	Gender, Race/ Ethnicity, Age, etc. in tabular format.
**Economic Impacts**	
Impact – Direct and Indirect	What economic impacts were discussed by the source, and how these were measured, categorised into direct and indirect impacts (tabular format)
Interventions	Where the source discusses the introduction of an intervention, what type of intervention it was, its comparator and the details of its introduction.
Other Key Findings	Any other key points related to the scoping review research questions.

Knowledge users and PPI representatives will be provided the opportunity to participate in Stage 3 and Stage 4 of the scoping review process. If interested in participating, they will receive training on the software used for data screening and extraction.

### Stage 5: Collating, summarising and reporting the results

Data charting will be completed using Covidence and will be shared among the team for review and sign off. The results of the review will be presented as a map of the data from the included studies according to the aims of the review, as recommended by the JBI Manual for Evidence Synthesis ( Peters *et al*., 2020a). This will involve two aspects: reporting of the overall results, which will be presented in tabular form and include aspects such as number of studies included, types of study design, etc; and a basic descriptive analysis, which will apply meaning to the results ( Levac *et al.*, 2010). Thematic analysis will not be undertaken as it is outside the remit of scoping reviews ( Peters *et al*., 2020a); however, a table according to the PAGER framework will be provided. This framework, which looks at the patterns (P), advances (A), gaps (G), Evidence for practice (E) and research recommendations (R), provides a consistent approach for the reporting of scoping review findings ( Bradbury-Jones *et al*., 2022). The analysis methodology will be reported to ensure transparency and rigour in the analysis process and assist in future replication. The results of the review will be validated through peer debriefing and PPI representative and knowledge user consultation, discussed in the next section. Gaps in the literature will be highlighted as areas for future research.

High levels of heterogeneity between contexts, impacts and diseases are anticipated. Reporting of the impacts and costs incurred by rare diseases in the included studies will depend on and be influenced by the rare diseases and various other factors, such as access to health services and social assistance. Results will be reported according to country (to reflect the differing health systems and socio-economic contexts) and according to disease groupings. Rare diseases will be categorised according to Orphanet groupings, which group rare diseases according to relevant medical specialties ( Rare Disease Research Landscape Steering Group, 2023).

### Stage 6: Consultation

This review will include consultation with two groups: PPI representatives and knowledge users. The PPI representatives include two mothers and two fathers who are parents of children and young people with rare diseases and who have experience with the economic impacts of rare diseases for families with single and multiple children with rare diseases. These PPI representatives are active in the rare disease community and were involved in the conceptualisation of the overall project.

The knowledge users linked with this study include medical paediatric consultants, policmakers, and representatives of advocacy organisations all working in the rare disease field. For the purposes of this review, a knowledge user is defined as:

*“one in a position of authority to influence and/or make decisions about health policy or the delivery of services and can act to ensure that the findings of the research will be translated to influence decision making and change within their (or other) organisations” ( Health Research Board, 2023, p.7).*

This scoping review protocol was presented to the PPI representatives and knowledge users working in the rare disease field and the research questions and search strategy were validated through separate discussion and consultation to understand the priorities of the two groups. Following completion of the data analysis of this review, the results of the review will be presented to the PPI representatives and knowledge users for validation and to ensure the implications of the findings are meaningful to their community. Feedback from the groups will be reported in and incorporated into the final review.

Dissemination of the research findings will also be developed through knowledge user engagement. The same knowledge users and PPI representatives will be invited to participate in the development of an evidence summary for dissemination on social media. This is to ensure knowledge translation and impact is accessible and meaningful to the communities it is targeted at and is in line with the guidance for knowledge user engagement developed by Pollock *et al*. (2022).

As the PRISMA-ScR tool currently does not include the reporting of knowledge user engagement ( Pollock *et al*., 2022), the participation of knowledge users in the consultation process will be reported using the ACTIVE framework, developed by Pollock *et al*. (2019) for use in systematic reviews. This framework is appropriate for use in this review due to the similarities in conduct processes between systematic and scoping reviews ( Pollock *et al*., 2022).

## Dissemination

The proposed scoping review will be submitted for publication in peer-reviewed academic journals. As previously discussed, an evidence summary will be developed for social media through knowledge user engagement, to ensure the meaningful transmission of results. In addition, the results will be presented at conferences and shared with relevant stakeholders and policymakers where appropriate.

## Study Status

This review is currently in the stage three: study selection phase. Database searches have been performed and the results are being screened by title and abstract by two reviewers.

## Conclusions

Rare diseases are associated with significant economic impacts for families of children living with rare diseases; however, the literature in this area is sparse and as a topic, it remains underexplored. The results of this scoping review will map existing work on the economic impacts of living with a rare disease, for children and their families, contributing to the literature on this topic. The results will also be used to develop a survey that will measure the economic and psychosocial costs of living with a rare disease in Northern Ireland and the Republic of Ireland.

## Data Availability

No data are associated with this article.
