# Supplementary material for: The economic impact of living with a rare disease for children and their families: a scoping review protocol
Source: HRB Open Res. 2024 Apr 8;6:41. Originally published 2023 Aug 31. [Version 2] doi: 10.12688/hrbopenres.13765.2 (PMC11187531; doi:10.12688/hrbopenres.13765.2)
Supplement: Peer review history

# Reviewer report: Reviewer response for version 2

**DOI:** 10.21956/hrbopenres.15178.r39811

**Published:** 2024-06-19

**Authors:** Genevieve Currie

**Affiliations:** 1 School of Nursing and Midwifery, Mount Royal University, Calgary, AB, Canada

Thank you for the opportunity to provide this review. This is a strong scoping review protocol paper. However, I have highlighted some things I think you could still focus on with in your paper to make your paper stronger.

1. Please do not use the word patient to describe a person with a rare disease. Use the word person or child instead as you are not describing someone receiving an intervention or care in this manuscript.
2. You could also include as therapy as direct costs therapy for children with rare diseases (physio, occupational, speech and behavioral), educational supports with aides and educational assistants and indirect costs for parents could be respite for caregiver support. I see some of these in table one but in the literature review these are not mentioned.
3. Since this scoping review is focused on children and their families, I think this should be explicitly stated early in the third paragraph of the literature review. The manuscript also describes costs for adults with rare diseases but this should be only for context and not figure so prominently in the literature review as this is not the focus of this paper.
4. I don’t understand research question #4: “To determine the study settings, rare conditions and geographical contexts, and the study types and organizations involved (e.g., charitable organisations, pharmaceutical companies, etc.). “What do you mean by determine study settings?  Are you asking to determine where other research studies have occurred and who was studied and who was involved in conducting the study? Just needs to be clearer. You could use the concepts you list under table 4 with charting the data.
5. You mention indirect costs to siblings in the context. I think you could add siblings earlier in the background when you say children and their families, including siblings as they are in important family members to understand the impacts.
6. Consultation: Will the PPI parents be parents of the same child i.e. a mother and father of the same child? I think it would increase diversity of the consultation if they were 4 parents of 4 different children. This could be stated in this section.

 

1. Consultation: policymakers is misspelled.
2. I would put this sentence earlier in the manuscript under methods to demonstrate you had engagement with representatives much earlier than the consultative stage: “This scoping review protocol was presented to the PPI representatives and knowledge users working in the rare disease field and the research questions and search strategy were validated through separate discussion and consultation to understand the priorities of the two groups.”
3. Well designed and supported from multiple frameworks and theories.
4. Excellent to include parents as consultants as well as knowledge users. Great to give the definition of a knowledge user that you are using.

Is the study design appropriate for the research question?

Yes

Is the rationale for, and objectives of, the study clearly described?

Partly

Are sufficient details of the methods provided to allow replication by others?

Yes

Are the datasets clearly presented in a useable and accessible format?

Yes

Reviewer Expertise:

NA

I confirm that I have read this submission and believe that I have an appropriate level of expertise to confirm that it is of an acceptable scientific standard.

# Reviewer report: Reviewer response for version 2

**DOI:** 10.21956/hrbopenres.15178.r39074

**Published:** 2024-04-13

**Authors:** Andrew Dwyer

**Affiliations:** 1 Boston College, Massachusetts, USA

The authors have responded appropriately to the points raised in the initial review.

Is the study design appropriate for the research question?

Yes

Is the rationale for, and objectives of, the study clearly described?

Yes

Are sufficient details of the methods provided to allow replication by others?

Partly

Are the datasets clearly presented in a useable and accessible format?

Not applicable

Reviewer Expertise:

Rare diseases

I confirm that I have read this submission and believe that I have an appropriate level of expertise to confirm that it is of an acceptable scientific standard.

# Reviewer report: Reviewer response for version 1

**DOI:** 10.21956/hrbopenres.15057.r36287

**Published:** 2023-10-24

**Authors:** Andrew Dwyer

**Affiliations:** 1 Boston College, Massachusetts, USA

Buckle and colleagues present a protocol for a scoping review that aims to map the current literature on the economic impact of pediatric rare diseases. Specifically the scoping review will examine how costs for pediatric rare diseases have been explored, what approaches have been employed, and what gaps remain in our understanding of the economic impact of rare pediatric conditions on families. This is a timely and important topic that merits investigation. A relative strength of the protocol include the rigor of the approach. Authors propose to: follow the PRISMA-ScR guidelines to guide the process, utilize the PCC model to guide article identification, search 6 databases, use supporting software (EndNote, Covidence) and have public and patient involvement (PPI). After carefully reviewing the protocol I have a number of questions that I feel merit consideration:

1. Authors appropriately cite the PRISMA-ScR yet I feel it would be useful to consider using an economic evaluation guideline from the EQUATOR network (i.e., CHEERS reporting guideline) to aid in identifying gaps in the literature and guide future reporting efforts for consistency and comparability.
2. The protocol describes cost of illness analysis as well as direct and indirect healthcare costs. Given that rare disease patients are geographically dispersed and have difficulty accessing specialized care I feel that noting travel costs is a relevant and key consideration.
3. The protocol cites recent economic evaluations for rare diseases (i.e., Delaye et al., 2022 and Garcia-Perez et al., 2021) yet it is not completely clear how the proposed scoping review will differ from the recent analysis or what gap this study will fill. Did these prior studies include (at least to some extent) a focus on pediatrics? This could be clarified for precision.
4. I applaud the PPI but there is relatively scant description of this. Have PPI stakeholders already been identified? If so, who are they and what perspectives do they bring? Similarly, it is not exactly clear at what stages they will be involved. Having a separate section detailing PPI would be beneficial. Similarly, who are the “knowledge users in clinical practice and policy” stakeholders?
5. When describing the population the term “economic impact” is not clearly defined. Is it direct costs? Indirect costs? Both? Other? Please clarify for precision.
6. The protocol correctly states that definitions of what makes a condition rare varies by country. I would appreciate more clarity on how rarity will be defined. Presumably by the country the investigators hail form. How will this be handled in international (cross-border) collaborative studies?
7. Under “types of sources” it is stated that “Results excluded due to language will be reported transparently”. This seems vague, please clarify.
8. I was confused by the inclusion criteria. Shouldn’t this include an inclusion criteria specifying articles that report on healthcare costs for rare diseases?
9. The “Stage 2: Identifying relevant studies” was not entirely clear. I understand that titles and abstracts as well as full text will undergo independent dual review. Will data extraction also be independently done by two investigators then compared?
10. In Table 2, please define abbreviations (i.e., S1, S2, S3, S4).
11. In the section on “Stage 5”, what does the sentence “Explicit details of the methodology for analysis will be provided to ensure transparency and rigour in the analysis process”? Would not this be part of the quality appraisal? Please clarify.
12. In Table 3, health finance and healthcare system structures vary widely between countries. For example, the U.S. has health insurance tied to employment with largely a fee-for-service structure, while the U.K. has nationalized healthcare (i.e., NHS) and Switzerland has a mandatory insurance program. I feel that it is important to extract and describe information on the respective health systems.
13. In the conclusions section it would be helpful to operationally define terminology that is introduced. Specifically, what is a “cost-minimal intervention”?

Is the study design appropriate for the research question?

Yes

Is the rationale for, and objectives of, the study clearly described?

Yes

Are sufficient details of the methods provided to allow replication by others?

Partly

Are the datasets clearly presented in a useable and accessible format?

Not applicable

Reviewer Expertise:

Rare diseases

I confirm that I have read this submission and believe that I have an appropriate level of expertise to confirm that it is of an acceptable scientific standard, however I have significant reservations, as outlined above.

# Response

**Published:** 2023-12-18

**Authors:** Niamh Buckle

We thank the reviewer for their comments and considered feedback on this scoping review protocol.

 We thank the reviewer for their comments and acknowledge their thoughts on the length and lack of highlighted focus in the introduction of the protocol. The introduction has been edited to clearly highlight how this scoping review differs from other scoping reviews on the topic.

 We acknowledge the lack of clarity provided on the possible development of a ‘cost-minimal’ intervention following completion of the scoping review as part of this multiphase project. Mention of a ‘cost-minimal’ intervention has been removed. The results of this review will instead inform further research on this topic.

 We thank the reviewer for acknowledging the strength of the scoping review through its reliance on methodology framework, and for their thoughtful comments on aspects we should consider further. We have incorporated the Consolidated Health Economic Evaluation Reporting Standards 2022 (CHEERS 2022) into the quality assessment of the included studies, instead of the MMAT tool. The PRISMA guidance will be retained, as it reports the scoping review process.

 We thank the reviewer for their comments and considered suggestions. More detail on the PPI aspects of the review have been provided. Further information on the PPI representatives and knowledge users is included in the Consultation section of the review. Information on the relationships between the PPI representatives and the research team is also provided.

 We thank the reviewer for their thoughts on methods, specifically around the unclear definition of ‘economic impact’. An additional table is provided with examples of direct and indirect healthcare and non-healthcare costs that will be used to illustrate economic impact.

 We thank the reviewer for their request for clarity on the inclusion and exclusion criteria. The vague statement has been addressed. Regarding the inclusion criteria, this paper will focus on studies considering all the costs associated with rare diseases, not only healthcare costs. The types of costs to be included have been more clearly defined. The wording has been changed regarding if any studies will be excluded due to language barriers. An additional table is provided with examples of direct and indirect healthcare and non-healthcare costs.

 We acknowledge the reviewer’s concerns around stage 5 of the review. As this is a scoping review, the method of analysis will not be as in-depth as a systematic review or meta-analysis. However, the analysis methodology will be reported to ensure transparency and rigour in the analysis process and assist in future replication.

 We thank the reviewer for their thoughts on the data extraction component of this review. Travel costs have been included in Table 1 of costs to be included. A section for defining the relevant health system for each included article has been included in the data extraction chart.

 We thank the reviewer for their request for clarity on how rarity will be defined. Rare diseases will be defined according to Orphanet classifications, which has been cross-referenced across contexts and databases, and therefore should be acceptable across any contexts included in the review.

 We thank the reviewer for their request for clarity on Stage 2. More information on the data extraction process has been provided to address their query.

 We thank the reviewer for their request for clarity on abbreviations included in Table 2. The abbreviations in the relevant table are now defined.

# Reviewer report: Reviewer response for version 1

**DOI:** 10.21956/hrbopenres.15057.r35969

**Published:** 2023-10-23

**Authors:** Yvonne Zurynski

**Affiliations:** 1 Professor of Health System Sustainability, Macquarie University, Sydney, New South Wales, Australia

An interesting protocol for a scoping review.

 The introduction is excessively long and reads like a literature review for a thesis rather than the background for a peer-reviewed publication. I would suggest shortening the background to include a very clear rationale that supports the need for this scoping review.

 In the abstract, the authors mention that the review will support a cost-minimal intervention - there is no rationale provided as to why this might be suggested before the review has even been done! If you are going to use terms such as "cost-minimal" these need to be clearly defined.

 I would suggest that the main research question is revised to include how the economic impacts are measured, the types and magnitude of these impacts rather than just how impacts are "identified". 

 The methods rely on the JBI 6-step process and PRISMA-ScR which is a good basis for this type of review.

 The involvement of patient and public is commendable, however, it's not clear whether the PPI panel has already been established, what kinds of representatives are included in the PPI and exactly how they have contributed to the development of this protocol - some additional clarification would be good to include.

 In the methods please provide clear definitions of direct and indirect costs that will be included and/or excluded in the review, is loss of income included or excluded? Is travel to appointments included or excluded? What about the cost of medical equipment (wheelchairs etc.) and medical consumables (e.g. dressings, oxygen tubing and masks, etc...).

 Please be clear on what study designs are included and excluded - for example the methods say that "case-control studies and analytical cross-sectional studies will also be considered" - it's not clear what this means - will you include such studies or exclude them?

 Justification to use the MMAT tool is required. Why are you not using quality assessment tools specifically developed for health economic studies e.g. CHEC, QHES or HEE?

 Stage 5 seems to have been a little glossed over when actually this is extremely important for a review such as this. It is very likely that there will be high levels of heterogeneity among the included studies and yet this is not addressed. For example the economic outcomes and impacts are likely to differ depending on the country of origin and factors such as the degree of universal coverage, access to private health insurance and social security supports will need to be considered - how will you deal with these very different socio-economic contexts?

 What exactly is meant by "financial experiences"?

 There is likely to be a significant level of heterogeneity of rare diseases included in the identified studies - what will be the strategy for interpreting the economic impact for families who have a child with for example a developmentally disabling disease such as Rett Syndrome compare with for example Fabry disease where the impacts are different?

 Including an empty PRISMA chart as a figure holds little value for the reader of this protocol.

 I would suggest that the authors think through a little bit more carefully about what details they may wish to extract in their data charting to ensure that they extract adequate detail for synthesis and interpretation.

Is the study design appropriate for the research question?

Partly

Is the rationale for, and objectives of, the study clearly described?

Yes

Are sufficient details of the methods provided to allow replication by others?

Partly

Are the datasets clearly presented in a useable and accessible format?

Not applicable

Reviewer Expertise:

Health services and systems research; models of care, impacts on families, chronic and complex disease including rare diseases

I confirm that I have read this submission and believe that I have an appropriate level of expertise to state that I do not consider it to be of an acceptable scientific standard, for reasons outlined above.

# Response

**Published:** 2023-12-18

**Authors:** Niamh Buckle

We thank the reviewer for their comments and considered feedback on this scoping review protocol.

 We acknowledge their thoughts on the length of the introduction of the protocol. The introduction has been edited for clarity as per the reviewer’s suggestion.

 We acknowledge the lack of clarity provided on the possible development of an intervention following completion of the scoping review as part of this multiphase project. Mention of a ‘cost-minimal’ intervention has been removed. The results of this review will instead inform further research on this topic.

 Following the reviewer’s suggestion, the research question has been amended to better reflect the aims of the review.

 We thank the reviewer for acknowledging the strength of the scoping review through its reliance on methodology framework, and for their thoughtful comments on aspects we should consider further. We have incorporated the Consolidated Health Economic Evaluation Reporting Standards 2022 (CHEERS 2022) into the quality assessment of the included studies, instead of the MMAT tool.

 We thank the reviewer’s for their comments and considered suggestions. More detail on the PPI aspects of the review have been provided, including further information on the PPI representatives and knowledge users, in the Consultation section of the review. Information on the relationships between the PPI representatives and the research team is also provided.

 We thank the reviewer for their thoughts on methods, specifically around the direct and indirect costs to be included, which was unclear. An additional table is provided with examples of direct and indirect healthcare and non-healthcare costs.

 We thank the reviewer for requesting clarity on the types of sources to be included. Additional text has been inserted clarifying the types of sources to be included. The focus of the review on any design that provide information on the economic costs (direct and/ or indirect) of paediatric rare diseases and meet the inclusion criteria is highlighted.

 We acknowledge the reviewer’s concerns around stage 5 and the likely high heterogeneity of included studies in the review. As this is a scoping review, the method of analysis will not be as in-depth as a systematic review or meta-analysis. Furthermore, the review does not aim to evaluate the economic impact of each rare disease, but rather understand what types of costs are evaluated in the included studies. The likely high levels of heterogeneity will be addressed following data extraction and analysis according to the articles retrieved. The data extraction table will include the country of origin of the articles, the rare diseases evaluated by the articles, and the types of costs evaluated by the articles, therefore allowing the mapping of these types of costs according to country and disease.

 We thank the reviewer for pointing out the lack of clarity of the term ‘financial experiences’. This term has been removed in favour of ‘direct and indirect costs’.

 We thank the reviewer for their comment on Figure 1. This figure has been removed.

 We thank the reviewer for their thoughts on the data extraction component of this review. The data extraction chart has been updated to address the reviewers’ concerns and provide further detail.
